# Crystal structure of (7-methyl-2-oxo-2*H*-chromen-4-yl)methyl piperidine-1-carbo­di­thio­ate

**DOI:** 10.1107/S2056989015013699

**Published:** 2015-07-29

**Authors:** K. R. Roopashree, T. G. Meenakshi, K. Mahesh Kumar, O. Kotresh, H. C. Devarajegowda

**Affiliations:** a Department of Physics, Yuvaraja’s College (Constituent College), University of Mysore, Mysore 570 005, Karnataka, India; b Department of Physics, Y.Y.D. Govt. First Grade College, Belur 573 115 Hassan, Karnataka, India; c Department of Chemistry, Karnatak University’s Karnatak Science College, Dharwad, Karnataka 580 001, India

**Keywords:** crystal structure, 2*H*-chromene, hydrogen bonding, C—H⋯π inter­actions, π–π inter­actions

## Abstract

In the title compound, C_17_H_19_NO_2_S_2_, the 2*H*-chromene ring system is nearly planar, with a maximum deviation of 0.0383 (28) Å, and the piperidine ring adopts a chair conformation. The 2*H*-chromene ring makes dihedral angles of 32.89 (16) and 67.33 (8)°, respectively, with the mean planes of the piperidine ring and the carbodi­thio­ate group. In the crystal, C—H⋯O and weak C—H⋯S hydrogen bonds link the mol­ecules into chains along [001]. The crystal structure also features C—H⋯π and π–π inter­actions, with a centroid–centroid distance of 3.7097 (17) Å.

## Related literature   

For biological applications of coumarins, see: Stiefel *et al.* (1995 ▸); Murray *et al.* (1982 ▸); Khan *et al.* (2004 ▸); Kawaii *et al.* (2001 ▸); Yu *et al.* (2003 ▸). For biological applications of di­thia­carbamates, see: D’hooghe & de Kime (2006 ▸); Thorn & Ludwig (1962 ▸); Cao *et al.* (2005 ▸). For a related structure, see: Kumar *et al.* (2013 ▸).
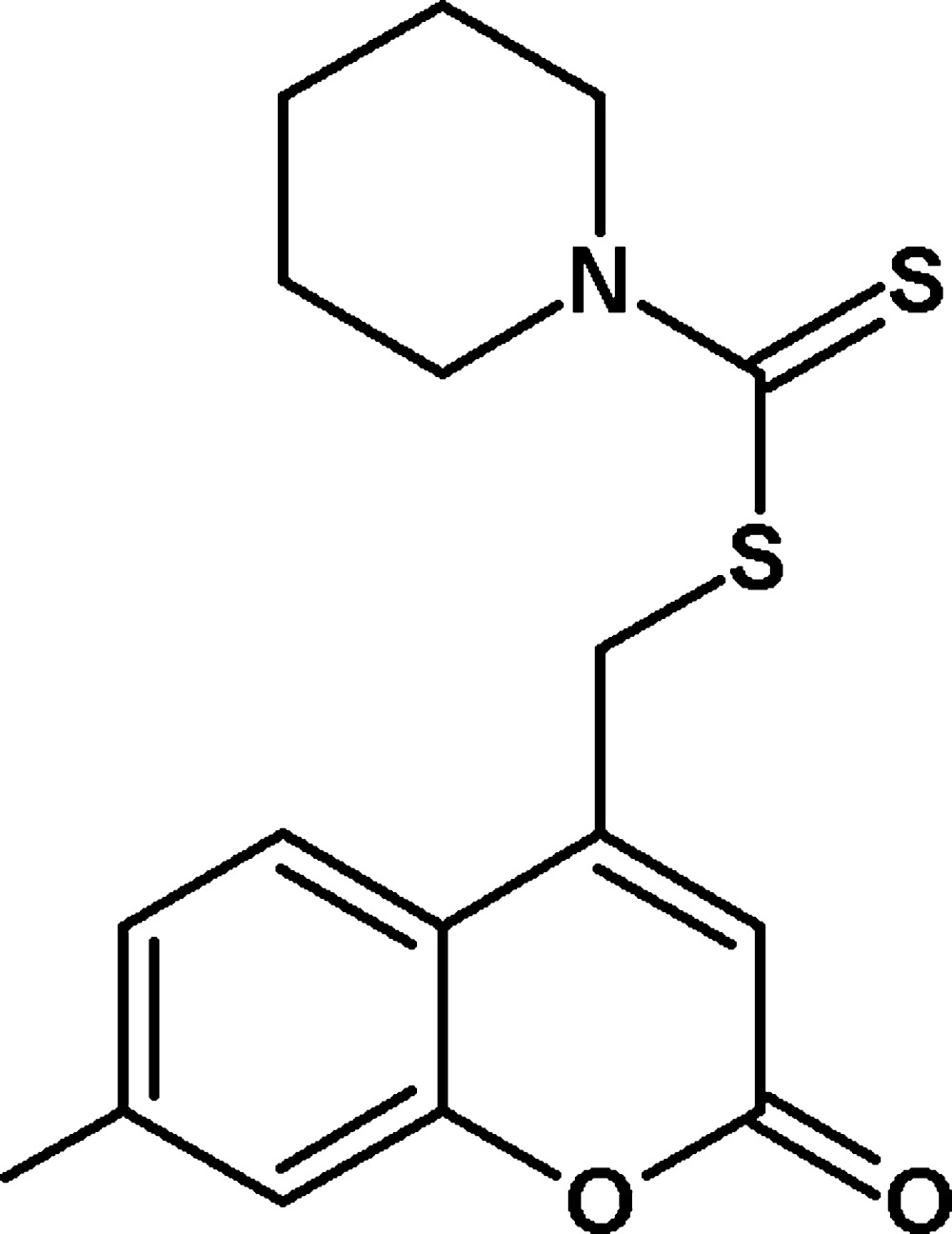


## Experimental   

### Crystal data  

C_17_H_19_NO_2_S_2_
*M* *_r_* = 333.45
Monoclinic, 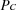

*a* = 4.9641 (2) Å
*b* = 11.4351 (3) Å
*c* = 14.0023 (4) Å
β = 90.743 (2)°
*V* = 794.77 (4) Å^3^
*Z* = 2
Mo *K*α radiation
μ = 0.34 mm^−1^
*T* = 296 K
0.24 × 0.20 × 0.12 mm

### Data collection  

Bruker SMART CCD area-detector diffractometer
Absorption correction: ψ scan (*SADABS*; Sheldrick, 2007 ▸) *T*_min_ = 0.770, *T*_max_ = 1.000
4426 measured reflections
2119 independent reflections
2027 reflections with *I* > 2σ(*I*)
*R*_int_ = 0.017

### Refinement  

*R*[*F* ^2^ > 2σ(*F* ^2^)] = 0.026
*wR*(*F* ^2^) = 0.062
*S* = 1.04
2119 reflections
200 parameters
2 restraints
H-atom parameters constrained
Δρ_max_ = 0.14 e Å^−3^
Δρ_min_ = −0.13 e Å^−3^
Absolute structure: Flack *x* determined using 704 quotients [(*I* ^+^)−(*I* ^−^)]/[(*I* ^+^)+(*I* ^−^)] (Parsons *et al.*, 2013 ▸)
Absolute structure parameter: 0.05 (3)

### 

Data collection: *SMART* (Bruker, 2001 ▸); cell refinement: *SAINT* (Bruker, 2001 ▸); data reduction: *SAINT*; program(s) used to solve structure: *SHELXS2014* (Sheldrick, 2008 ▸); program(s) used to refine structure: *SHELXL2014* (Sheldrick, 2015 ▸); molecular graphics: *ORTEP-3 for Windows* (Farrugia, 2012 ▸); software used to prepare material for publication: *SHELXL2014*.

## Supplementary Material

Crystal structure: contains datablock(s) I, global. DOI: 10.1107/S2056989015013699/zl2626sup1.cif

Structure factors: contains datablock(s) I. DOI: 10.1107/S2056989015013699/zl2626Isup2.hkl

Supporting information file. DOI: 10.1107/S2056989015013699/zl2626Isup3.cml

**Fig. 1.**
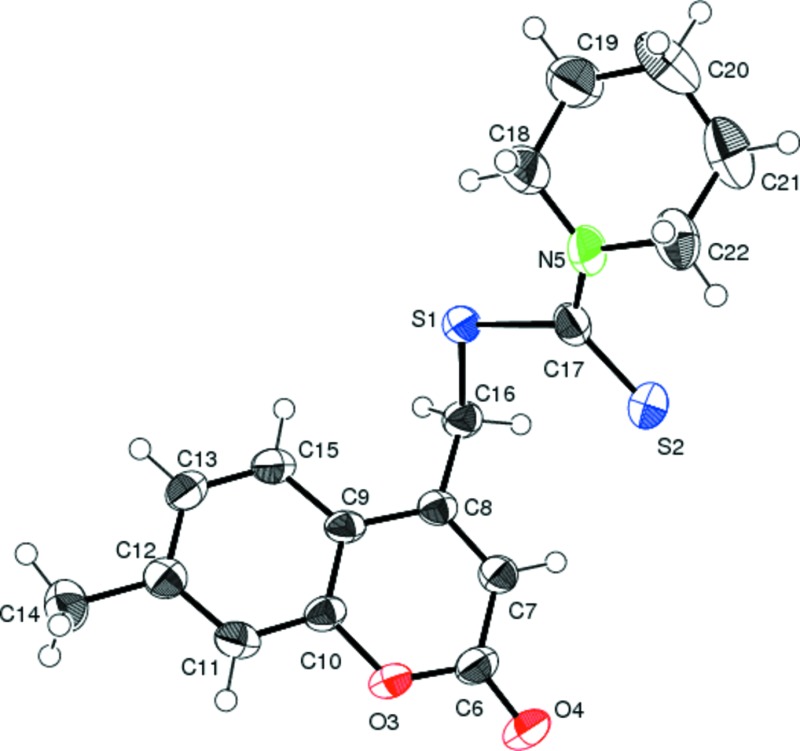
The molecular structure of the title compound. Displacement ellipsoids are drawn at the 50% probability level. Hydrogen atoms are shown as spheres of arbitrary radius.

. DOI: 10.1107/S2056989015013699/zl2626fig1.tif
The mol­ecular structure of the title compound. Displacement ellipsoids are drawn at the 50% probability level. Hydrogen atoms are shown as spheres of arbitrary radius.

**Fig. 2.**
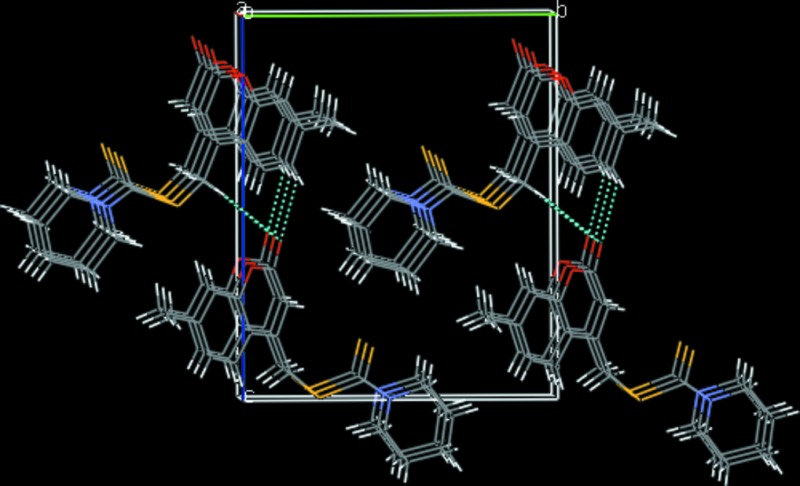
Crystal packing for the title compound with hydrogen bonds drawn as dashed lines.

. DOI: 10.1107/S2056989015013699/zl2626fig2.tif
Crystal packing for the title compound with hydrogen bonds drawn as dashed lines.

CCDC reference: 1413856

Additional supporting information:  crystallographic information; 3D view; checkCIF report

## Figures and Tables

**Table 1:** Hydrogen-bond geometry (, )

*D*H*A*	*D*H	H*A*	*D* *A*	*D*H*A*
C13H13O4^i^	0.93	2.45	3.223(4)	140
C16H16*B*S2	0.97	2.70	3.152(4)	109
C18H18*B*S1	0.97	2.38	2.930(4)	116
C22H22*A*S2	0.97	2.55	3.065(4)	113

Symmetry code: (i) 
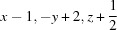
.

## supplementary crystallographic information

### S1. Comment

Coumarins and their derivatives play an important role in the agricultural and pharmaceutical industries (Stiefel *et al.*, 1995). They are widely present in higher plants such as Rutaceae, Apiaceae, Asteraceae, Leguminosae, Thymelaeaceae, and they also occur as animal and microbial metabolites (Murray *et al.*, 1982). Most of them show a wide spectrum of pharmacological effects, including antimicrobial (Khan *et al.*, 2004), anti-arrhythmic, antiosteoporosis, anti-HIV, and antitumor activities (Kawaii *et al.*, 2001; Yu *et al.*, 2003). Accordingly, many reports have described various structures and biological evaluations of numerous coumarin analogs newly synthesized or isolated from plants.

Sulfur containing molecules are currently under study as chemoprotectants in chemotherapy. Organic substances with a dithio functional group have been widely used in industry as rodent repellents, vulcanization additives in rubber manufacturing, additives in lubricants, and in agriculture as fungicides on almond trees, stone fruits, and vegetables. Among the various sulfur ligands being examined currently, dithiocarbamates have a special significance owing to their many uses, *e.g.* in analytical determinations, as arrestors of human immunodeficiency virus infections such as AIDS, in pharmaceutical products, in agriculture as pesticides and fungicides, and as high-pressure lubricants (D'hooghe & de Kime, 2006; Thorn & Ludwig, 1962; Cao *et al.*, 2005). One molecule of (7-methyl-2-oxo-2*H*-chromen-4-yl)methylpiperidine-1-carbodithioate is shown in Fig. 1. The 2*H*chromene ring system (O3/C6–C13/C15) is essentially planar, with a maximum deviation of 0.0383 (28) Å for atom C10 and the piperidine (N5/C18–C22) ring adopts a chair conformation. The dihedral angle of the 2*H*-chromene (O3/C6–C13/C15) ring with the piperidine (N5/C18–C22) ring and carbodithioate group are 32.89 (16)° and 67.33 (8)°, respectively. In addition, intermolecular C—H···O and weak C—H···S hydrogen bonds (Table 1) link the components into chains along [001]. The crystal structure also features C—H···π [C_g_(3) (C9–C15)] and [C_g_(1) (O3/C6–C10)]π–π [C_g_(3) (C9–C15)] interactions, with centroid–centroid distances of 3.7081 (15) Å that further stabilize the crystal packing, Figure 2.

### S2. Experimental

The title compound compound was prepared according to a reported method (Kumar *et al.*, 2013). Colourless needles of the title compound were grown from a mixed solution of EtOH/CHCl_3_ (*v*/*v* = 1/1) by slow evaporation at room temperature. Yield: 80%, m.p. 420 K.

### S3. Refinement

All H atoms were positioned geometrically, with C—H = 0.93 Å for aromatic H, C—H = 0.97 Å for methylene H and C—H = 0.96 Å for methyl H,and refined using a riding model with *U*_iso_(H) = 1.5*U*_eq_(C) for methyl H and *U*_iso_(H) = 1.2*U*_eq_(C) for all other H.

### Crystal data

**Table:**

C_17_H_19_NO_2_S_2_	*D*_x_ = 1.393 Mg m^−^^3^
*M**_r_* = 333.45	Melting point: 420 K
Monoclinic, *P**c*	Mo *K*α radiation, λ = 0.71073 Å
*a* = 4.9641 (2) Å	Cell parameters from 2119 reflections
*b* = 11.4351 (3) Å	θ = 1.8–25.0°
*c* = 14.0023 (4) Å	µ = 0.34 mm^−^^1^
β = 90.743 (2)°	*T* = 296 K
*V* = 794.77 (4) Å^3^	Plate, colourless
*Z* = 2	0.24 × 0.20 × 0.12 mm
*F*(000) = 352	

### Data collection

**Table:**

Bruker SMART CCD area-detector diffractometer	2119 independent reflections
Radiation source: fine-focus sealed tube	2027 reflections with *I* > 2σ(*I*)
Graphite monochromator	*R*_int_ = 0.017
Detector resolution: 10.0 pixels mm^-1^	θ_max_ = 25.0°, θ_min_ = 1.8°
ω and φ scans	*h* = −5→5
Absorption correction: ψ scan (*SADABS*; Sheldrick, 2007)	*k* = −13→13
*T*_min_ = 0.770, *T*_max_ = 1.000	*l* = −13→16
4426 measured reflections	

### Refinement

**Table:**

Refinement on *F*^2^	Hydrogen site location: inferred from neighbouring sites
Least-squares matrix: full	H-atom parameters constrained
*R*[*F*^2^ > 2σ(*F*^2^)] = 0.026	*w* = 1/[σ^2^(*F*_o_^2^) + (0.0331*P*)^2^ + 0.0991*P*] where *P* = (*F*_o_^2^ + 2*F*_c_^2^)/3
*wR*(*F*^2^) = 0.062	(Δ/σ)_max_ < 0.001
*S* = 1.04	Δρ_max_ = 0.14 e Å^−^^3^
2119 reflections	Δρ_min_ = −0.13 e Å^−^^3^
200 parameters	Absolute structure: Flack *x* determined using 704 quotients [(*I*^+^)-(*I*^-^)]/[(*I*^+^)+(*I*^-^)] (Parsons *et al.*, 2013)
2 restraints	Absolute structure parameter: 0.05 (3)

### Special details

**Table:**

Geometry. All esds (except the esd in the dihedral angle between two l.s. planes) are estimated using the full covariance matrix. The cell esds are taken into account individually in the estimation of esds in distances, angles and torsion angles; correlations between esds in cell parameters are only used when they are defined by crystal symmetry. An approximate (isotropic) treatment of cell esds is used for estimating esds involving l.s. planes.

### Fractional atomic coordinates and isotropic or equivalent isotropic displacement parameters (Å^2^)

**Table:**

	*x*	*y*	*z*	*U*_iso_*/*U*_eq_	
S1	0.15995 (16)	0.77643 (6)	0.48353 (6)	0.0417 (2)	
S2	0.42720 (17)	0.60022 (8)	0.35570 (7)	0.0529 (3)	
O3	0.1614 (4)	0.99061 (19)	0.14352 (14)	0.0405 (5)	
O4	0.4912 (5)	0.8912 (2)	0.07897 (17)	0.0579 (7)	
N5	0.0507 (6)	0.5536 (2)	0.4834 (2)	0.0496 (7)	
C6	0.3753 (7)	0.9153 (3)	0.1512 (2)	0.0414 (8)	
C7	0.4412 (7)	0.8712 (3)	0.2455 (2)	0.0379 (8)	
H7	0.5761	0.8152	0.2518	0.045*	
C8	0.3150 (6)	0.9079 (2)	0.3245 (2)	0.0324 (7)	
C9	0.1022 (6)	0.9951 (2)	0.3146 (2)	0.0320 (7)	
C10	0.0312 (6)	1.0321 (2)	0.2230 (2)	0.0331 (7)	
C11	−0.1659 (6)	1.1147 (3)	0.2057 (2)	0.0373 (7)	
H11	−0.2102	1.1357	0.1433	0.045*	
C12	−0.2979 (6)	1.1663 (2)	0.2814 (2)	0.0370 (7)	
C13	−0.2277 (6)	1.1305 (3)	0.3735 (2)	0.0396 (8)	
H13	−0.3149	1.1640	0.4252	0.047*	
C14	−0.5104 (7)	1.2582 (3)	0.2648 (3)	0.0509 (9)	
H14A	−0.5426	1.2672	0.1975	0.076*	
H14B	−0.4501	1.3312	0.2913	0.076*	
H14C	−0.6742	1.2348	0.2952	0.076*	
C15	−0.0339 (6)	1.0474 (2)	0.3902 (2)	0.0366 (7)	
H15	0.0076	1.0254	0.4526	0.044*	
C16	0.4049 (6)	0.8639 (3)	0.4206 (2)	0.0379 (7)	
H16A	0.4527	0.9305	0.4601	0.045*	
H16B	0.5665	0.8174	0.4125	0.045*	
C17	0.2071 (6)	0.6311 (3)	0.4400 (2)	0.0378 (7)	
C18	−0.1184 (8)	0.5752 (3)	0.5662 (3)	0.0561 (10)	
H18A	−0.3046	0.5577	0.5499	0.067*	
H18B	−0.1071	0.6570	0.5839	0.067*	
C19	−0.0287 (10)	0.5001 (3)	0.6495 (3)	0.0669 (11)	
H19A	0.1498	0.5243	0.6704	0.080*	
H19B	−0.1507	0.5116	0.7022	0.080*	
C20	−0.0229 (10)	0.3718 (3)	0.6234 (3)	0.0746 (13)	
H20A	−0.2056	0.3438	0.6136	0.090*	
H20B	0.0579	0.3275	0.6754	0.090*	
C21	0.1378 (9)	0.3527 (3)	0.5330 (3)	0.0733 (14)	
H21A	0.3265	0.3689	0.5461	0.088*	
H21B	0.1224	0.2716	0.5136	0.088*	
C22	0.0400 (8)	0.4302 (3)	0.4529 (3)	0.0630 (11)	
H22A	0.1521	0.4192	0.3974	0.076*	
H22B	−0.1436	0.4095	0.4353	0.076*	

### Atomic displacement parameters (Å^2^)

**Table:**

	*U*^11^	*U*^22^	*U*^33^	*U*^12^	*U*^13^	*U*^23^
S1	0.0590 (5)	0.0291 (4)	0.0373 (4)	0.0030 (4)	0.0125 (4)	0.0021 (4)
S2	0.0684 (6)	0.0435 (5)	0.0469 (5)	0.0125 (4)	0.0112 (5)	−0.0040 (4)
O3	0.0532 (14)	0.0428 (13)	0.0256 (12)	0.0032 (10)	0.0063 (10)	0.0005 (9)
O4	0.0800 (17)	0.0586 (16)	0.0356 (14)	0.0054 (14)	0.0226 (13)	−0.0038 (11)
N5	0.0614 (18)	0.0287 (13)	0.0591 (19)	0.0034 (12)	0.0108 (16)	−0.0015 (12)
C6	0.055 (2)	0.0355 (17)	0.034 (2)	−0.0090 (16)	0.0097 (17)	−0.0066 (14)
C7	0.0457 (19)	0.0311 (16)	0.0370 (19)	−0.0016 (14)	0.0064 (16)	0.0018 (13)
C8	0.0378 (17)	0.0284 (15)	0.0311 (17)	−0.0092 (13)	0.0014 (14)	0.0009 (12)
C9	0.0389 (17)	0.0287 (15)	0.0285 (18)	−0.0074 (13)	0.0054 (14)	0.0013 (12)
C10	0.0429 (17)	0.0304 (15)	0.0262 (16)	−0.0078 (13)	0.0048 (14)	−0.0022 (12)
C11	0.0458 (19)	0.0343 (16)	0.0319 (18)	−0.0061 (15)	−0.0001 (15)	0.0054 (13)
C12	0.0410 (17)	0.0295 (16)	0.0407 (19)	−0.0082 (13)	0.0063 (15)	0.0018 (14)
C13	0.0464 (19)	0.0342 (16)	0.038 (2)	−0.0052 (15)	0.0136 (16)	−0.0059 (13)
C14	0.049 (2)	0.0407 (19)	0.063 (2)	0.0030 (16)	0.0030 (19)	0.0030 (16)
C15	0.0479 (18)	0.0354 (16)	0.0266 (16)	−0.0073 (15)	0.0026 (15)	0.0009 (13)
C16	0.0450 (19)	0.0350 (16)	0.0338 (18)	−0.0026 (14)	0.0008 (15)	−0.0004 (14)
C17	0.0439 (18)	0.0316 (16)	0.0377 (19)	0.0058 (14)	−0.0067 (15)	−0.0004 (13)
C18	0.061 (2)	0.040 (2)	0.068 (3)	0.0003 (17)	0.014 (2)	0.0126 (17)
C19	0.082 (3)	0.055 (2)	0.064 (3)	−0.003 (2)	−0.001 (2)	0.0077 (19)
C20	0.085 (3)	0.048 (2)	0.090 (4)	−0.011 (2)	−0.035 (3)	0.024 (2)
C21	0.073 (3)	0.038 (2)	0.109 (4)	−0.001 (2)	−0.026 (3)	−0.002 (2)
C22	0.075 (3)	0.0324 (18)	0.081 (3)	−0.0030 (18)	0.003 (2)	−0.0062 (18)

### Geometric parameters (Å, º)

**Table:**

S1—C17	1.786 (3)	C13—H13	0.9300
S1—C16	1.812 (3)	C14—H14A	0.9600
S2—C17	1.657 (3)	C14—H14B	0.9600
O3—C6	1.370 (4)	C14—H14C	0.9600
O3—C10	1.378 (3)	C15—H15	0.9300
O4—C6	1.202 (4)	C16—H16A	0.9700
N5—C17	1.330 (4)	C16—H16B	0.9700
N5—C18	1.461 (4)	C18—C19	1.511 (5)
N5—C22	1.475 (4)	C18—H18A	0.9700
C6—C7	1.447 (4)	C18—H18B	0.9700
C7—C8	1.346 (4)	C19—C20	1.512 (6)
C7—H7	0.9300	C19—H19A	0.9700
C8—C9	1.458 (4)	C19—H19B	0.9700
C8—C16	1.499 (4)	C20—C21	1.521 (6)
C9—C10	1.392 (4)	C20—H20A	0.9700
C9—C15	1.397 (4)	C20—H20B	0.9700
C10—C11	1.379 (4)	C21—C22	1.505 (6)
C11—C12	1.385 (4)	C21—H21A	0.9700
C11—H11	0.9300	C21—H21B	0.9700
C12—C13	1.394 (5)	C22—H22A	0.9700
C12—C14	1.505 (4)	C22—H22B	0.9700
C13—C15	1.371 (5)		
			
C17—S1—C16	104.81 (15)	C8—C16—H16A	108.4
C6—O3—C10	121.6 (2)	S1—C16—H16A	108.4
C17—N5—C18	126.5 (3)	C8—C16—H16B	108.4
C17—N5—C22	121.6 (3)	S1—C16—H16B	108.4
C18—N5—C22	111.9 (3)	H16A—C16—H16B	107.5
O4—C6—O3	117.2 (3)	N5—C17—S2	125.3 (2)
O4—C6—C7	125.6 (3)	N5—C17—S1	112.6 (2)
O3—C6—C7	117.1 (3)	S2—C17—S1	122.13 (19)
C8—C7—C6	122.6 (3)	N5—C18—C19	110.5 (3)
C8—C7—H7	118.7	N5—C18—H18A	109.6
C6—C7—H7	118.7	C19—C18—H18A	109.6
C7—C8—C9	118.7 (3)	N5—C18—H18B	109.6
C7—C8—C16	119.8 (3)	C19—C18—H18B	109.6
C9—C8—C16	121.5 (3)	H18A—C18—H18B	108.1
C10—C9—C15	116.7 (3)	C20—C19—C18	111.8 (4)
C10—C9—C8	118.0 (3)	C20—C19—H19A	109.3
C15—C9—C8	125.3 (3)	C18—C19—H19A	109.3
C11—C10—O3	115.7 (3)	C20—C19—H19B	109.3
C11—C10—C9	122.7 (3)	C18—C19—H19B	109.3
O3—C10—C9	121.6 (3)	H19A—C19—H19B	107.9
C10—C11—C12	120.0 (3)	C19—C20—C21	110.6 (3)
C10—C11—H11	120.0	C19—C20—H20A	109.5
C12—C11—H11	120.0	C21—C20—H20A	109.5
C11—C12—C13	117.9 (3)	C19—C20—H20B	109.5
C11—C12—C14	121.2 (3)	C21—C20—H20B	109.5
C13—C12—C14	120.9 (3)	H20A—C20—H20B	108.1
C15—C13—C12	121.9 (3)	C22—C21—C20	111.6 (4)
C15—C13—H13	119.1	C22—C21—H21A	109.3
C12—C13—H13	119.1	C20—C21—H21A	109.3
C12—C14—H14A	109.5	C22—C21—H21B	109.3
C12—C14—H14B	109.5	C20—C21—H21B	109.3
H14A—C14—H14B	109.5	H21A—C21—H21B	108.0
C12—C14—H14C	109.5	N5—C22—C21	109.7 (3)
H14A—C14—H14C	109.5	N5—C22—H22A	109.7
H14B—C14—H14C	109.5	C21—C22—H22A	109.7
C13—C15—C9	120.9 (3)	N5—C22—H22B	109.7
C13—C15—H15	119.6	C21—C22—H22B	109.7
C9—C15—H15	119.6	H22A—C22—H22B	108.2
C8—C16—S1	115.4 (2)		
			
C10—O3—C6—O4	−173.9 (3)	C14—C12—C13—C15	−179.7 (3)
C10—O3—C6—C7	6.4 (4)	C12—C13—C15—C9	0.1 (5)
O4—C6—C7—C8	175.3 (3)	C10—C9—C15—C13	0.2 (4)
O3—C6—C7—C8	−5.1 (4)	C8—C9—C15—C13	178.6 (3)
C6—C7—C8—C9	0.4 (4)	C7—C8—C16—S1	−115.5 (3)
C6—C7—C8—C16	−176.3 (3)	C9—C8—C16—S1	67.9 (3)
C7—C8—C9—C10	3.0 (4)	C17—S1—C16—C8	85.8 (3)
C16—C8—C9—C10	179.6 (3)	C18—N5—C17—S2	171.3 (3)
C7—C8—C9—C15	−175.4 (3)	C22—N5—C17—S2	−6.1 (5)
C16—C8—C9—C15	1.2 (4)	C18—N5—C17—S1	−7.9 (4)
C6—O3—C10—C11	174.8 (3)	C22—N5—C17—S1	174.7 (3)
C6—O3—C10—C9	−3.2 (4)	C16—S1—C17—N5	176.6 (2)
C15—C9—C10—C11	−1.0 (4)	C16—S1—C17—S2	−2.7 (2)
C8—C9—C10—C11	−179.6 (3)	C17—N5—C18—C19	−118.1 (4)
C15—C9—C10—O3	176.8 (2)	C22—N5—C18—C19	59.5 (4)
C8—C9—C10—O3	−1.7 (4)	N5—C18—C19—C20	−55.0 (5)
O3—C10—C11—C12	−176.5 (2)	C18—C19—C20—C21	51.6 (5)
C9—C10—C11—C12	1.5 (4)	C19—C20—C21—C22	−52.7 (5)
C10—C11—C12—C13	−1.1 (4)	C17—N5—C22—C21	117.6 (4)
C10—C11—C12—C14	178.9 (3)	C18—N5—C22—C21	−60.2 (4)
C11—C12—C13—C15	0.3 (4)	C20—C21—C22—N5	56.3 (4)

### Hydrogen-bond geometry (Å, º)

**Table:**

*D*—H···*A*	*D*—H	H···*A*	*D*···*A*	*D*—H···*A*
C13—H13···O4^i^	0.93	2.45	3.223 (4)	140
C16—H16*B*···S2	0.97	2.70	3.152 (4)	109
C18—H18*B*···S1	0.97	2.38	2.930 (4)	116
C22—H22*A*···S2	0.97	2.55	3.065 (4)	113

Symmetry code: (i) *x*−1, −*y*+2, *z*+1/2.
