# Supplementary figures and images for: Crystal structure of (7-methyl-2-oxo-2H-chromen-4-yl)methyl piperidine-1-carbo­di­thio­ate
Source: Acta Crystallogr E Crystallogr Commun. 2015 Jul 29;71(Pt 8):o606–7. doi: 10.1107/S2056989015013699 (PMC4571421; doi:10.1107/S2056989015013699)

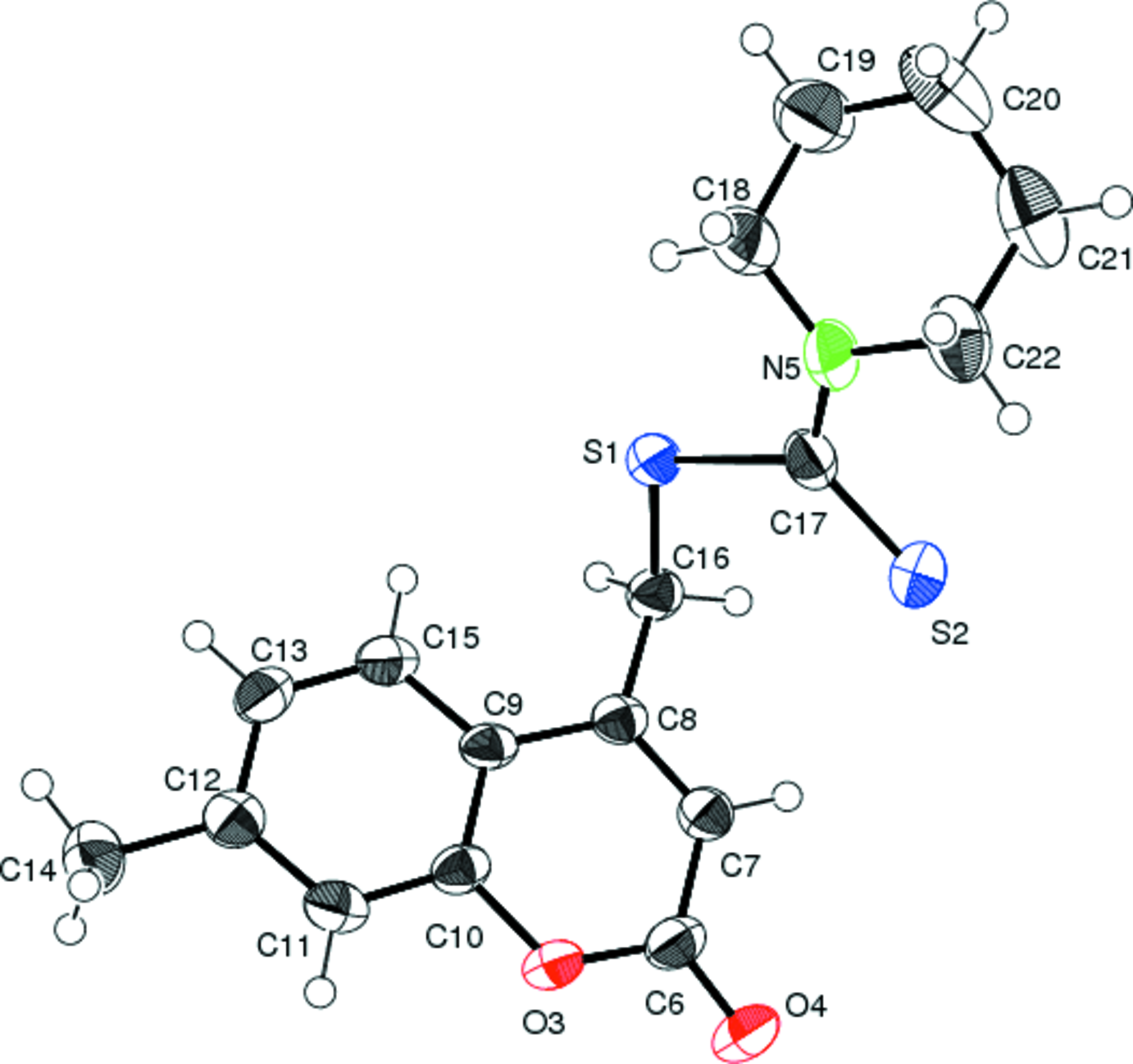

Supplement: Supplementary file 4 [file e-71-0o606-fig1.tif]

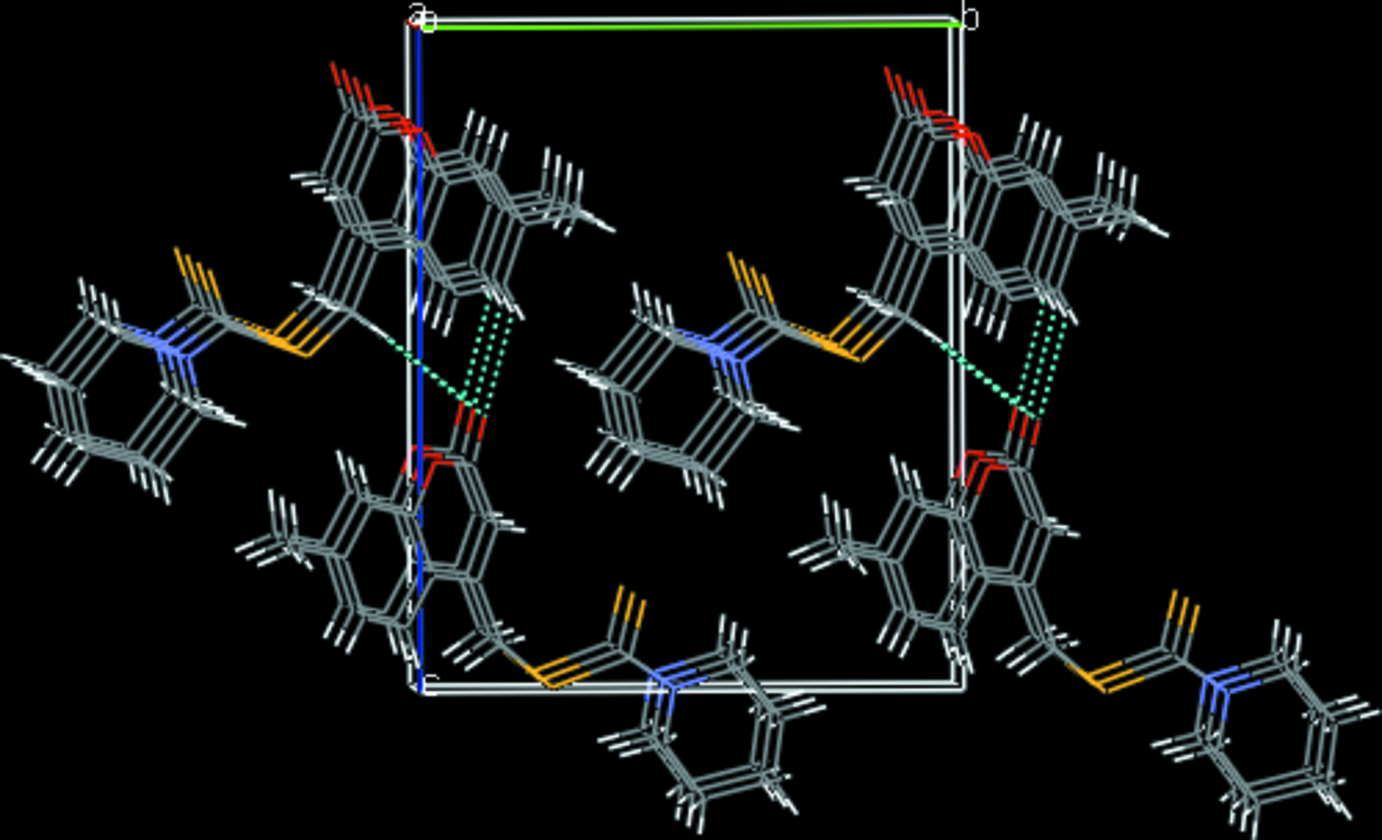

Supplement: Supplementary file 5 [file e-71-0o606-fig2.tif]
